# Impact of Macro-Polypropylene Fiber on the Mechanical Properties of Ultra-High-Performance Concrete

**DOI:** 10.3390/polym17091232

**Published:** 2025-04-30

**Authors:** Tamer Birol, Alper Avcıalp

**Affiliations:** 1 Department of Civil Engineering, Balıkesir University, 10145 Balıkesir, Türkiye; 2 Institute of Science, Balıkesir University, 10145 Balıkesir, Türkiye; alperavcialp@gmail.com

**Keywords:** ultra-high-performance concrete, polypropylene fiber, flexural tensile strength, digital image correlation, fib Model Code 2020

## Abstract

Steel fibers are frequently used in ultra high-performance concrete (UHPC) due to their superior properties, but they also have disadvantages, such as corrosion exposure, high specific gravity and high cost. Although synthetic fibers have emerged as an alternative, the focus has generally been on hybrid use with steel fibers in UHPC. This study investigates the applicability of macro-polypropylene (PP) fibers for UHPC in terms of mechanical properties. An experimental campaign was conducted for UHPC mixtures containing macro-PP fibers with varying volumetric ratios. The effects of macro-PP fiber on the mechanical properties of UHPC were investigated in terms of compressive strength, splitting tensile strength and flexural behavior. The two-dimensional digital image correlation (2D-DIC) method was adopted to examine the cracking behavior. In addition, tensile constitutive law for UHPC mixtures was obtained with inverse analysis based on Model Code 2020 (MC2020). The results showed that the use of macro-PP fibers had no significant impact on compressive and splitting tensile strength. However, residual flexural tensile strength and fracture energy increased by up to 2.8 and 2.5 times, respectively, compared to UHPC without fiber. It was determined that macro-PP fibers could exhibit effective crack control in UHPC.

## 1. Introduction

Ultra-high-performance concrete (UHPC) is a new-generation concrete with superior mechanical properties, such as very high compressive strength, improved post-cracking tensile behavior, and excellent durability [1,2,3,4,5]. However, the inclusion of fibers is crucial for UHPC to achieve its superior mechanical properties. Fibers bridge cracks within the concrete matrix, enabling stress transfer across the crack interfaces (crack bridging ability) and inhibiting further propagation. This mechanism enhances post-cracking behavior and leads to load bearing and energy absorption capacity under various loading conditions [6,7,8]. Although there are different types of fibers, high-strength steel fibers are mostly used in UHPC because of their improved crack-bridging ability, which contributes to the strain capacity of concrete under both compression and tensile effects [3,9,10,11]. It has been observed that steel fibers perform better in terms of the mechanical properties of UHPC such as compressive strength, tensile strength, flexural behavior, and ductility compared to other fiber types due to their high strength and modulus of elasticity [12,13,14,15]. Although steel fibers are widely used in UHPC, they have disadvantages such as corrosion exposure, high specific gravity, and high cost [16,17,18]. The steel fibers on the surface layer of the UHPC members can be affected by corrosion which leads to deterioration in mechanical properties [19,20]. Kim et al. [21] stated that the cost of steel fiber at 1% by volume is higher than the cost of the entire mixture. In addition, steel is classified as one of the materials of which the production is harmful to the environment and causes high greenhouse gas emissions [22,23,24]. Due to these disadvantages, the use of synthetic fibers such as an alternative to steel fibers in fibrous concrete has been the subject of many research studies [25,26,27,28,29].

The most common types of synthetic fibers utilized in fibrous concrete are carbon-based polymeric fibers, including polypropylene (PP), polyethylene (PE), and polyvinyl alcohol (PVA) [30]. Synthetic fibers, typically derived from synthesized polymers through chemical extraction techniques, are characterized by high corrosion resistance, low weight, and reduced cost compared to steel fibers [31,32,33,34,35]. Synthetic fibers generally have lower tensile strength and modulus of elasticity than steel fibers. Therefore, they have limited contribution to the compressive strength and elastic modulus of UHPC compared to steel fibers [36,37]. Furthermore, an increase in the amount or length of fibers can lead to the clustering of fibers, resulting in the reduced workability of concrete, localized weak areas, and strain softening behavior [4,29,38,39]. However, studies have shown that synthetic fibers significantly increase the ductility, toughness, and freeze–thaw resistance of concrete, while also reducing shrinkage, crack width and spalling during fire [29,40,41,42]. Synthetic fibers can be classified into two categories: micro-fibers and macro-fibers. These distinctions are based on the diameter of the fibers, with micro-fibers having a diameter less than 0.3 mm and macro-fibers having a diameter greater than 0.3 mm [43]. Synthetic fibers are often mixed with other fiber types, especially steel fibers, in UHPC [41,44,45,46,47,48,49,50]. Incorporating hybrid fibers in UHPC aims to achieve a synergistic effect of different fibers to enhance overall performance. These investigations observed that hybridization provides advantages in strength, ductility, and durability.

Polypropylene (PP) fibers are the most commonly used synthetic fibers in cement-based materials because of their resistance to a wide range of chemical agents, inert hydrophobic nature, thermal stability, high melting point, lower cost, and ease of production. PP fibers are generally considered to have a lower modulus of elasticity compared to other fiber types [51,52,53]. Studies have shown that macro-PP fibers increase the post-crack strength and energy absorption capacity of concrete [25,54,55,56]. Since the modulus of elasticity of PP fibers is lower than that of steel fibers (4 to 10 GPa), they need to be used in high proportions to achieve performances similar to steel fibers [57]. Hannawi et al. [38] found that the compressive strength of UHPC mixture without fiber decreased from 141 MPa to 124.7 MPa with the use of 1.0% macro-PP–PE fiber by volume. Li et al. [39] found that the flowability of UHPC increased with the increasing diameter of PP fibers, and decreased with increasing length and volume ratio. The study showed that increases in PP fiber length and dosage had a much stronger effect on permeability than increases in fiber diameter. The significant improvement was reported in Al-Mwanes and Aghayari [58] for the splitting tensile strength and flexural strength of UHPC containing micro-PP fibers compared to UHPC without fibers. He et al. [41] found that high-performance macro-PP fibers showed an improved toughening effect for UHPC. The findings demonstrate that a 2.0% fiber content in UHPC is associated with enhanced mechanical properties. Neira Medina et al. [44] conducted a comparative study on the impact of nylon, PVA, PP, PP-PE and steel fibers on the flexural behavior of UHPC. Among synthetic fibers, the UHPC mixture containing 2% macro-PP fibers by volume ratio exhibited the most favorable properties in fresh and hardened states. According to Yan et al. [37], when the dosage of micro-PP fibers was increased, the fluidity and compressive strength of UHPC mixtures decreased compared to the non-fiber UHPC mixture while the flexural strength increased. Shen et al. [42] reported that the fluidity, compressive strength and elastic modulus of UHPC decreased with the increasing volumetric ratio of micro-PP. However, micro-PP fibers improved the splitting tensile strength, axial tensile strength, and flexural toughness of UHPC. Lin et al. [45] found that the workability of UHPC was enhanced with the proportion of steel fibers replaced by PP fibers increased. The incorporation of low-modulus and low-strength PP fibers into the composition of UHPC resulted in a notable decline in both compressive and tensile strength. The study by Yassin et al. [59] indicated that PP fibers increased both the shear capacity and toughness of high-performance concrete (HPC) beams, particularly at a volume ratio of 1%.

A review of the existing studies shows that the research on the use of polypropylene (PP) fibers in UHPC is quite limited, while most of them focus on the use of PP fibers in combination with different fiber types. For hybrid fiber combinations, PP fibers are mostly combined with steel fibers. Very limited results are available in these studies on macro-PP fiber use alone. The findings of the studies demonstrated that PP fibers can provide benefits for UHPC in terms of mechanical properties. Nevertheless, further investigation is necessary to clarify the effects of macro-PP fiber use. In this study, the usability of macro-PP fiber in UHPC and its influence on mechanical properties were experimentally investigated. The study aimed to determine the effects of macro-PP fibers with respect to mechanical properties by considering different volumetric ratios. The mechanical properties of UHPC were investigated in terms of compressive strength, splitting tensile strength and flexural behavior. The two-dimensional digital image correlation (2D-DIC) method was adopted to examine the cracking pattern observed in the bending tests. In addition, tensile constitutive law for UHPC mixtures was obtained with inverse analysis based on MC2020 [60].

## 2. Experimental Design

**Figure 1:**
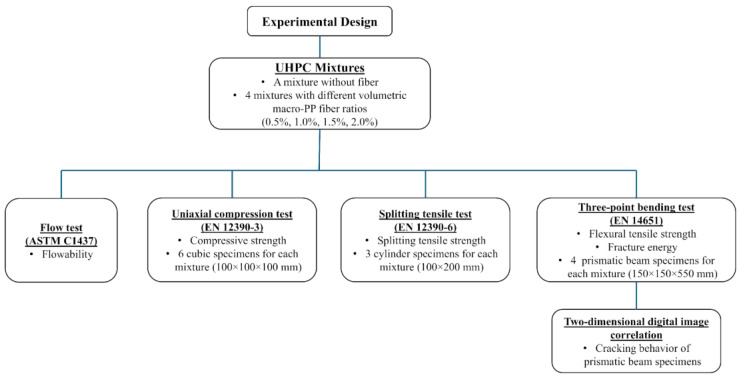
Schematic presentation of the experimental study.

The design of the experimental study, including mixture design variables, specimen details, testing procedures and investigation parameters, is illustrated in Figure 1.

### 2.1. Materials and Mixture Proportions

**Figure 2:**
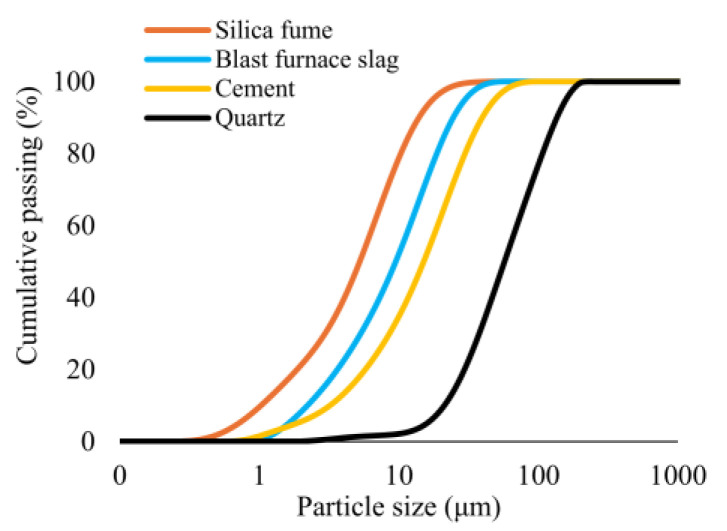
Particle size distribution of the materials.

**Figure 3:**
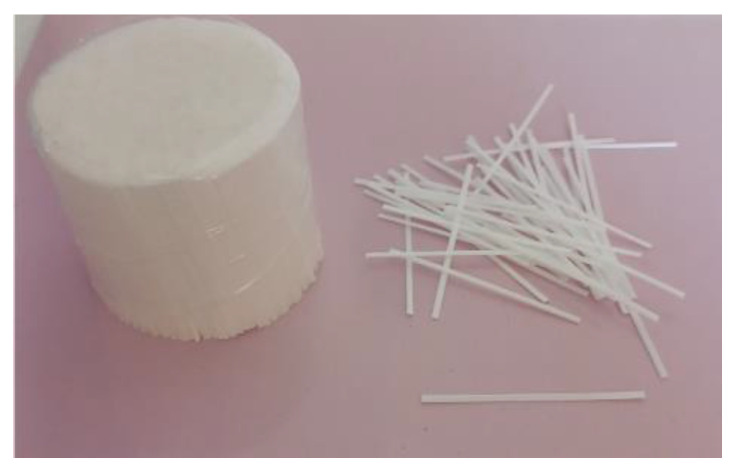
Macro-polypropylene fibers used in the study.

The mixture design for the UHPC utilized in this study was based on the methodology established by Hasgul et al. [61] and Turker et al. [62]. The base mixture included CEM I 42.5 Portland cement (Bursa Çimento, Bursa, Türkiye), silica fume (Finnfjord AS, Finnfjord, Norway), blast furnace slag (Oyak Bolu Çimento/Ankara, Türkiye), quartz sand (Aydınlar Madencilik/Izmir, Türkiye). The particle size distribution of the materials is shown in Figure 2 [63]. A high-range water-reducing admixture (ViscoCrete^®^ ACE 450) was included in mixtures to achieve proper workability without experiencing bleeding and segregation. The water-to-binder ratio was kept constant at 0.17 by weight. The maximum particle size of the quartz sand was 0.225 mm. The macro-polypropylene (PP) fibers (KORDSA/Istanbul, Türkiye) were considered in monofilament form, with a length of 48 mm and a thickness of 0.72 mm were considered (Figure 3). The polypropylene fibers have a tensile strength of 550 MPa and an elastic modulus of 8.5 GPa. The materials and proportions of the UHPC mixtures are given in Table 1 [63]. The NF indicated the reference UHPC mixture without fibers, whereas the letter F was used for UHPC mixtures with fibers. Four different volumetric ratios were considered for the UHPC mixtures with macro-PP fibers. The fiber content by volume is indicated with a number in the case of the letter. For example, F0.5 represents a UHPC mixture containing 0.5% macro-PP fiber by volume (Table 1).

### 2.2. Specimen Properties and Preparation

**Figure 4:**
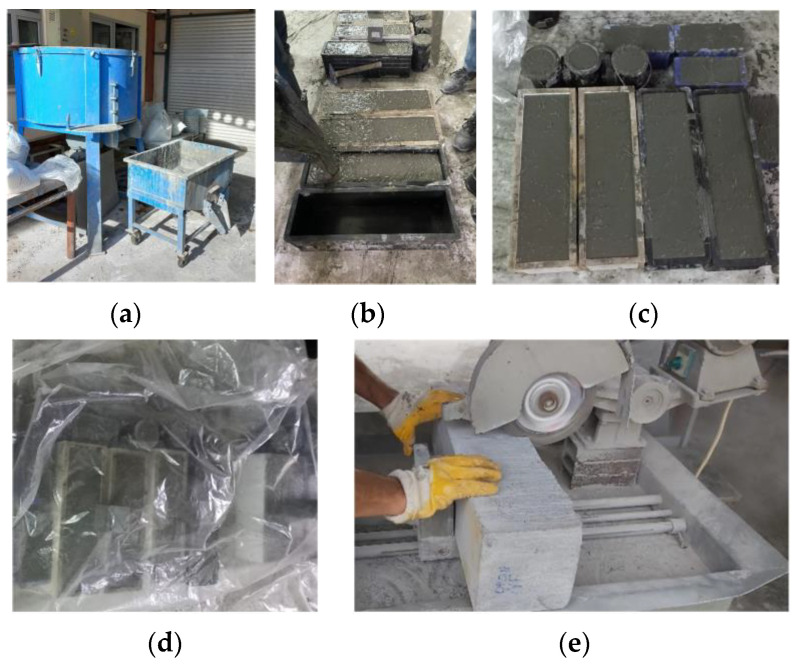
Preparation of test specimens: (**a**) concrete mixing setup; (**b**) casting; (**c**) specimens after casting; (**d**) curing; (**e**) notch sawing.

The locally manufactured pan-type concrete mixer with 100 L mixing capacity was used to produce UHPC mixtures (Figure 4a). The cement, silica fume, and blast furnace slag were premixed for 3 min. After that, the water and the admixture were added, and the mixing process continued. After obtaining a liquid consistency, the aggregate was included in the mixture and mixing was continued for about 5 min. Finally, the macro-PP fibers were gradually added to the mixture, avoiding agglomeration. After reaching considerable fluidity and viscosity, the UHPC was transferred from the mixer to the casting device, and concrete pouring was carried out by free flowing from one end of the molds (Figure 4b) [63]. In order to determine the compressive and splitting tensile strengths of UHPC mixtures, cubic specimens with 100 × 100 × 100 mm and cylinder specimens with dimensions of 100 × 200 mm (diameter × length) were prepared according to EN 12390-1 [64]. The flexural behavior was investigated by three-point bending tests on prismatic beam specimens with a cross-sectional dimension of 150 × 150 mm and a total length of 550 mm, in accordance with EN 14651 [65]. The test specimens after concrete casting are presented in Figure 4c. The molds were covered with a plastic sheet to prevent water evaporation and stored at room temperature for 24 h (Figure 4d). After demolding, the specimens were stored in room condition until the test day. The beams were turned on their sides at 90° to the casting surface, and a 25 mm deep, 2 mm wide notch was cut in the midspan (Figure 4e) [63].

### 2.3. Test Methods and Instrumentation

**Figure 5:**
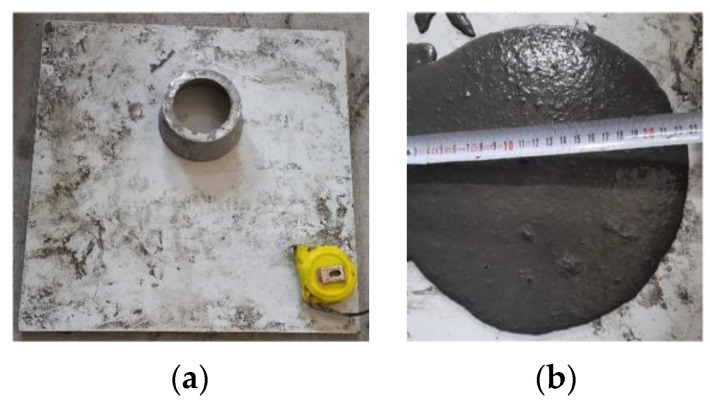
(**a**) Flow test setup; (**b**) spread measurement.

The flowability of the UHPC mixtures was determined during the production of the specimens by conducting a flow test according to ASTM C1437 [66]. After the concrete was placed in the mini cone, it was lifted vertically and allowed to spread freely. The spread value was measured in two perpendicular directions and averaged (Figure 5).

**Figure 6:**
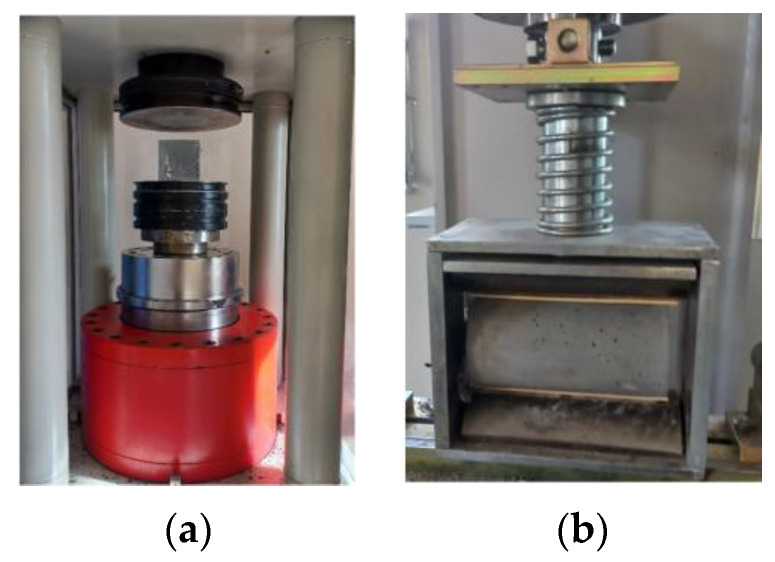
(**a**) Uniaxial compression test setup; (**b**) splitting tensile test setup.

The compressive strength of the mixtures was determined by the uniaxial compression tests of cubic specimens according to EN 12390-3 [67]. The tests were carried out under load control using a servo-hydraulic compression testing machine with a load capacity of 2500 kN (Dotek/Türkiye) (Figure 6a). The splitting tensile strength of the mixtures was determined by splitting tensile tests on cylinder specimens according to EN 12390-6 [68]. Cylinder test specimens were placed horizontally on the loading frame and loaded linearly in the perpendicular direction to the cross-section plane (Figure 6b). The constant loading rate was applied as 1256 N/sec [68].

**Figure 7:**
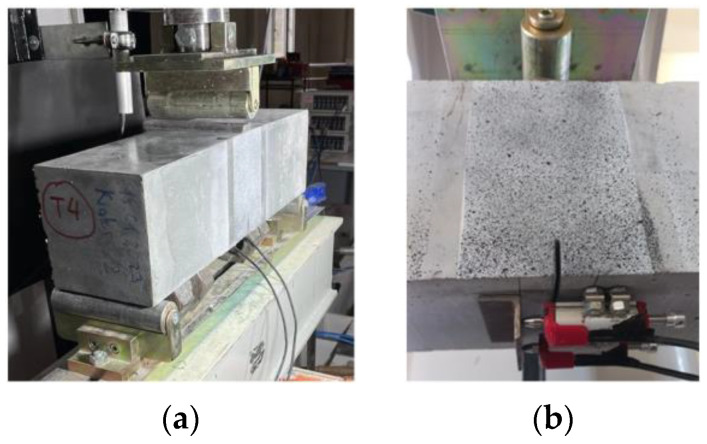
(**a**) Three-point bending setup; (**b**) CMOD setup.

To evaluate the effectiveness of macro-PP fibers on the flexural behavior of UHPC, three-point bending tests were performed on notched beam specimens according to EN 14651 [65]. The tests were carried out at a displacement rate of 0.2 mm/min using an actuator controlled by a servo hydraulic control unit with capacity of a 500 kN (Utest/Türkiye) (Figure 7a). The beams were simply supported with a clear span of 500 mm and the load was applied at the center of the span. Two 5 mm capacity linear transducers were placed horizontally on the bottom of the beam to determine the crack mouth opening displacement (CMOD) values which express the opening at the end of the notch located in the center of the span. An L-shaped steel plate was placed at the end of the transducers (Figure 7b) [63].

**Figure 8:**
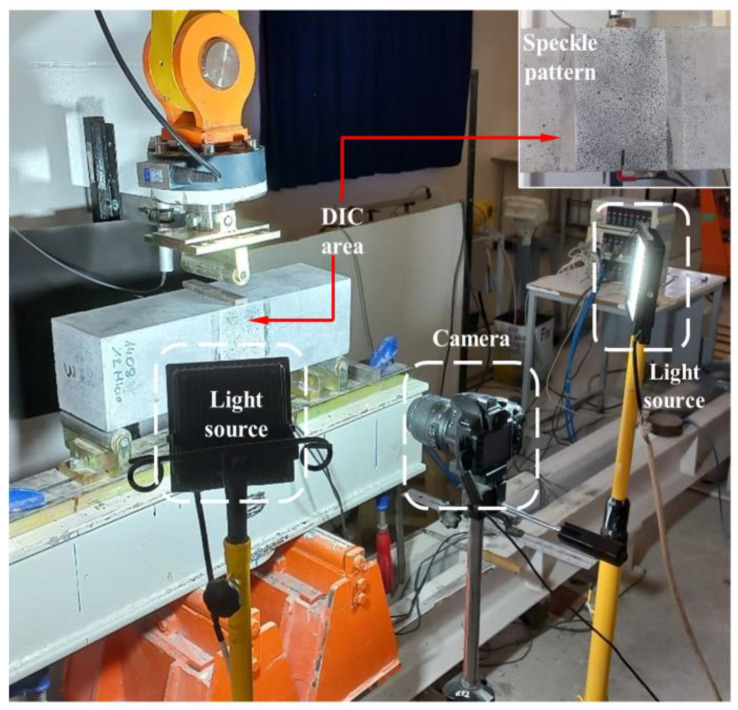
The 2D-DIC setup.

To investigate the effects of different macro-PP fiber ratios on the formation and development of cracks, the strain profile on the surface of the beams was monitored by the two-dimensional digital image correlation (2D-DIC) method. Since the crack will start at the notch, an area of 100 × 150 mm in the midspan of the beam covering the notch was chosen for 2D-DIC analysis. To conduct the 2D-DIC, it is necessary to apply a randomly distributed speckle pattern on the surface with high contrast. For this, the target area was sprayed with matte white paint. After the surface completely dried, the area was randomly spotted with matte black paint. A high-resolution camera, fixed on a tripod 50 cm away from the test specimen, was used to capture the images of the target area (Figure 8) [63]. To eliminate the differences between the images caused by variations in natural light, the target area was covered by two LED spotlights placed at an angle of 45° (Figure 8). During the tests, images with a resolution of 3008 × 2000 pixels were captured every 10 s. The 2D-DIC analyses of the images were performed with the GOM Correlate software [69] using the default correlation method. The subset size was selected as 19 × 19 pixels and the step size as 16 pixels.

## 3. Experimental Results

### 3.1. Fluidity of UHPC Mixtures

**Figure 9:**
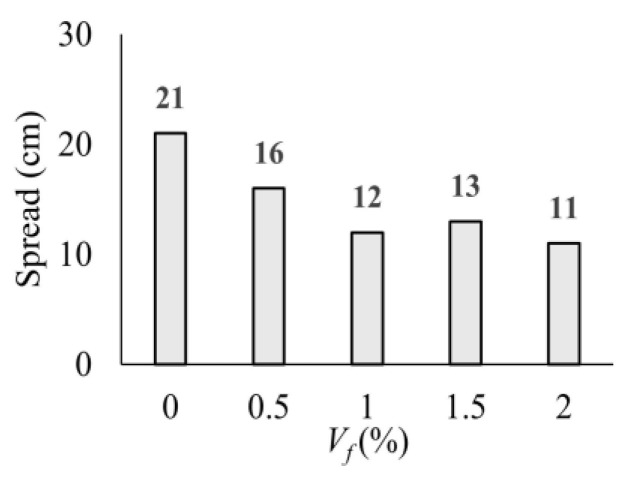
Spread values of UHPC mixtures with different fiber ratios.

The measured spread values for the UHPC mixtures with different volumetric fiber ratios (*V_f_*) are presented in Figure 9. The highest spreading value was obtained in the mixture NF as expected. It is clearly seen that the fluidity of UHPC decreases with increasing macro-PP fiber content. The spread value exhibited a notable decline compared to the NF mixture, reaching 24%, 43%, 38%, and 48% reductions for mixtures F0.5, F1.0, F1.5, and F2.0, respectively (Figure 9). It should be noted that the amount of high-range water-reducer additive was kept constant to investigate the effect of fiber ratio. The admixture content was slightly increased for the mixtures of 1.5% and 2.0% fiber by volume to prevent excessive agglomeration of fibers. However, at fiber contents above 0.5%, fluidity decreased significantly while spread values remained similar.

### 3.2. Compressive Strength

**Figure 10:**
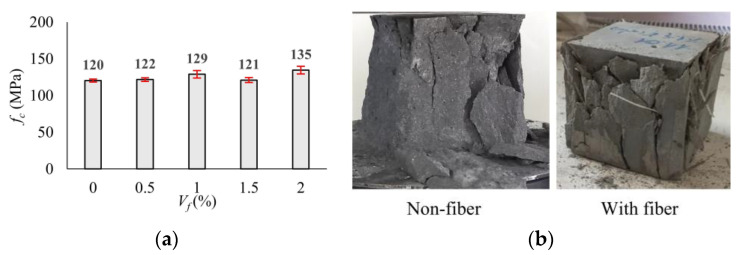
(**a**) Variation in the compressive strength with volumetric fiber ratio; (**b**) influence of macro-PP fiber on the fracture mode of cubic specimens.

The compressive strengths (*f_c_*) were determined by averaging the test results of the six cubic specimens for each mixture. The variation in the mean compressive strengths according to the volumetric fiber ratio (*V_f_*) is presented in Figure 10a. The comparative fracture modes of the non-fiber and fibrous specimens are shown in Figure 10b. While the tests of non-fiber specimens were terminated with a sudden explosion, the integrity of the specimens was maintained in the fibrous specimens (Figure 10b). It was seen that there is no significant difference in compressive strength for mixtures except mixture F2.0 containing 2.0% macro-PP fiber by volume. The maximum average compressive strength of 135 MPa was achieved for the mixture F2.0.

### 3.3. Splitting Tensile Strength

**Figure 11:**
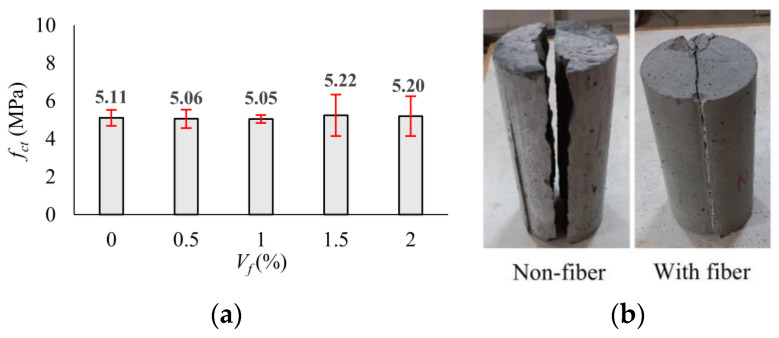
(**a**) Variation in splitting tensile strength with volumetric fiber ratio; (**b**) influence of macro-PP fiber on the fracture mode of cylinder specimens.

The fracture loads of the three cylinder specimens for each mixture were averaged, and the mean splitting tensile strengths (*f_ct_*) were calculated using Equation (1) provided in EN 12390-6 [68]. In Equation (1), *P* is the fracture load, *l* and *D* define the specimen length and diameter, respectively. The variation in the mean *f_ct_* values with volumetric fiber ratio (*V_f_*) is compared graphically in Figure 11a. There is no significant difference compared to the mixture NF for low fiber ratios (*V_f_* = 0.5% and 1.0%). At higher fiber ratios (1.5% and 2.0%), only an average increase of 2% could be achieved. It is seen that the use of macro-PP fiber has no significant contribution in terms of splitting tensile strength. As expected, the NF specimens were fractured into two pieces upon reaching the fracture load. However, it was observed that even at the lowest fiber content (0.5%), polypropylene fibers exhibited crack bridging ability (Figure 11b).

(1) $$f_{ct}=\frac{2\times P}{\pi \times l\times D}$$

### 3.4. Flexural Behavior

#### 3.4.1. Fracture Energy

**Figure 12:**
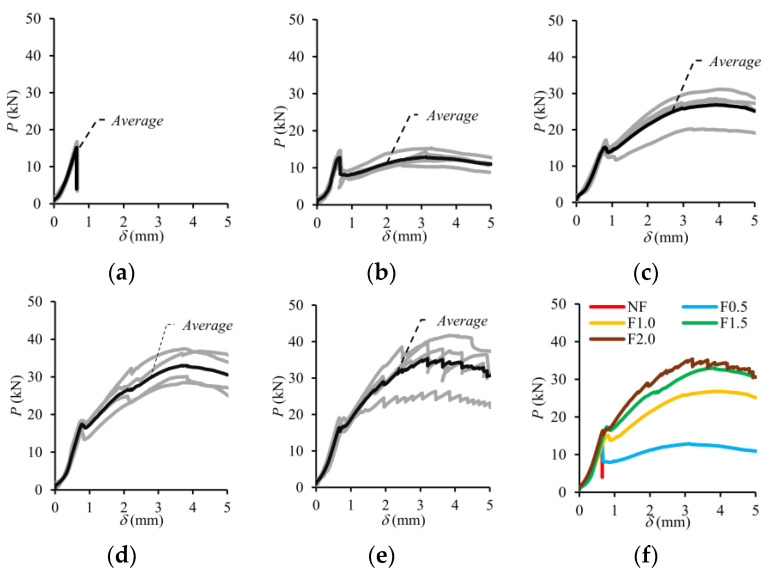
Experimental *P*-*δ* curves of the test beams: (**a**) *V_f_* = 0%; (**b**) *V_f_* = 0.5%; (**c**) *V_f_* = 1.0%; (**d**) *V_f_* = 1.5%; (**e**) *V_f_* = 2.0%; (**f**) average.

Fracture energy (*G_f_*) is defined as the energy required to produce a unit crack in the specimen. It is one of the fundamental properties of concrete that can be used to analyze and determine the toughness, brittleness, and crack resistance. The fracture energy can be calculated by Equation (2) according to RILEM 50-FMC [70]. The *w*_0_ is the area under the load–deflection (*P*-*δ*) curve (N/m), *m* is the mass (kg) of the beam specimens between the supports, *g* is the acceleration of gravity (m/s^2^), *b* is width of the cross-section and *h_sp_* is the distance between the tip of the notch and the top of the cross-section. The experimental *P*-*δ* relationships of the UHPC mixtures were obtained considering four beams for each mixture and presented in Figure 12. The average *P*-*δ* behaviors of the mixtures were also received and plotted with bold curves in Figure 12. The maximum deflection value (*δ_max_*) was limited to 5 mm depending on the capacity of the transducers. The average *P*-*δ* behaviors of the mixtures are comparatively shown in Figure 12f. In this study, the fracture energy was calculated for the areas up to the maximum load after cracking (*G_f,max_*) and up to 5 mm at which the tests were terminated (*G_f,_*_5mm_). Since the test was completed due to a sudden load drop after cracking in the non-fiber specimens (NF), no energy dissipation occurred after this point (Figure 12a). Incorporating macro-PP fiber into UHPC led to a notable improvement in post-peak behavior and, consequently, an enhancement of the overall energy absorption capacity (Figure 12b–e). This conclusion is consistent with the findings of previous studies [37,41,42].

(2) $$G_{f}=\frac{w_{0}+m\cdot g\cdot \delta }{b\cdot h_{sp}}$$

**Figure 13:**
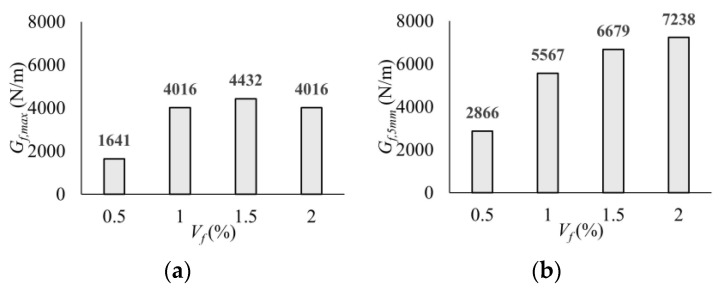
Fracture energy values at different stages: (**a**) *G_f,max_*; (**b**) *G_f,_*_5mm_.

Figure 13 graphically shows the variation in the *G_f_* value according to the volumetric fiber ratio (*V_f_*). The *G_f,max_* was obtained as 1641 N/m for a 0.5% fiber ratio, and an increase of up to 2.7 times was obtained with the increase in fiber content (Figure 13a). In the region up to the deflection value of 5 mm, where the tests were terminated, the absorbed energy (*G_f,_*_5mm_) increased to 7238 N/m due to the use of 2.0% fiber by volume with the effect of deflection hardening behavior. This value is 2.5 times higher than the *G_f,_*_5mm_ value obtained for 0.5% fiber usage (Figure 13b). It was observed that the macro-PP fiber significantly increased the ductility of UHPC, while the effect of 0.5% fiber by volume was limited compared to other fiber contents. It can be said that the optimum fiber content for the ductility parameter was determined to be 1.5% by volume (Figure 13).

#### 3.4.2. Load–Crack Mouth Opening Displacement Behavior

**Figure 14:**
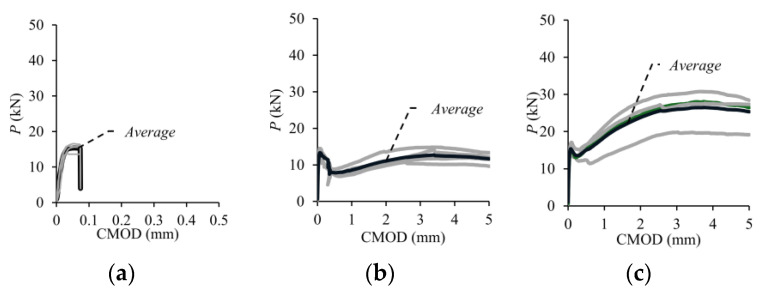

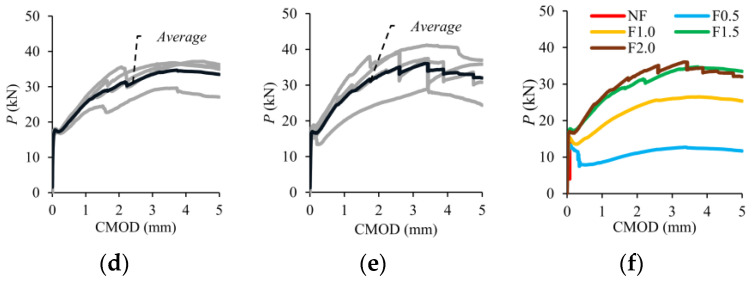
Experimental *P*-CMOD responses: (**a**) *V_f_* = 0%; (**b**) *V_f_* = 0.5%; (**c**) *V_f_* = 1.0%; (**d**) *V_f_* = 1.5%; (**e**) *V_f_* = 2.0%; (**f**) average.

The experimental load–crack mouth opening displacement (*P*-CMOD) responses of the four notched beams for each volumetric fiber ratio (*V_f_*) are presented in Figure 14, while the average curve is shown by bold lines. The average *P*-CMOD responses for each *V_f_* are also presented in Figure 14f. As seen in Figure 14, the load increased approximately linear with the increase in CMOD until cracking. When the cracking occurred, the elastic deformation phase ended. In the beams without fibers, deformation capacity was reached with the crack initiation (Figure 14a). On the other hand, the behavior of the beams with macro-PP fibers continued despite a sudden load drop depending on fiber ratio (Figure 14b–e).

The residual flexural tensile strengths (*f_R_*) obtained by using Equation (3) for certain values of CMOD to determine the characteristics of the post-cracking behavior according to EN 14651 [65]. In Equation (3), *P* is the load, *l* is the length between supports, *b* is the width of the beam cross-section, and *h_sp_* is the distance between the tip of the notch and the top of the cross-section.

**Figure 15:**
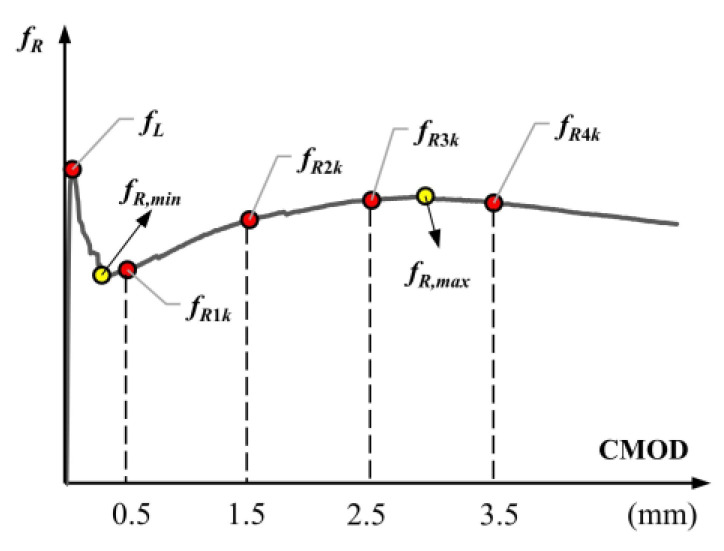
Characteristic points of the flexural tensile strength–CMOD curve.

In MC2020 [60], the characteristic residual strengths *f_R_*_1*k*_ (CMOD = 0.5 mm) for the serviceability limit state (SLS) and *f_R_*_3*k*_ (CMOD = 2.5 mm) for the ultimate limit state (ULS) are considered to classify the post-cracking strength of fibrous concrete. The strength at the limit of proportionality, corresponding to the onset of cracking in the concrete, is denoted as *f_L_*. The strengths corresponding to 1.5 mm and 3.5 mm CMOD values are denoted as *f_R_*_2*k*_ and *f_R_*_4*k*_, respectively [60]. The study also considered the minimum post-cracking strength following the load drop (*f_R,min_*) and the maximum post-cracking strength (*f_R,max_*). The characteristic strengths are shown schematically in Figure 15. The flexural strengths of the mixtures calculated using the average *P*-CMOD curves are presented in Table 2.

(3) $$f_{R}=\frac{3P\cdot l}{2b\cdot h_{sp}^{2}}$$

A sudden load drop was observed following the onset of cracking in all UHPC beams (Figure 14). However, it was observed that the load recovery after cracking was related to the volumetric fiber ratio. The *f_R,min_*/*f_L_* ratios for 0.5%, 1.0%, 1.5%, and 2.0% fiber contents by volume were determined as 0.56, 0.88, 0.97, and 0.97, respectively. These results show that a significant strength loss occurred after cracking for 0.5% macro-PP fiber use. On the other hand, a very quick load recovery was observed for other fiber ratios. The maximum post-cracking strength (*f_R,max_*) was below the cracking strength (*f_L_*) for the mixture F0.5, indicating the deflection softening behavior (Figure 14b). For all other fiber ratios, *f_R,max_* values exceeded *f_L_* values, and deflection hardening behavior was obtained (Figure 14c–e). In terms of hardening behavior, *f_R,max_*/*f_L_* ratios were determined as 1.73, 1.96, and 2.12 for 1.0%, 1.5%, and 2.0% fiber contents, respectively. It was observed that there was no significant difference between 1.5% and 2.0% macro-PP fiber use.

**Figure 16:**
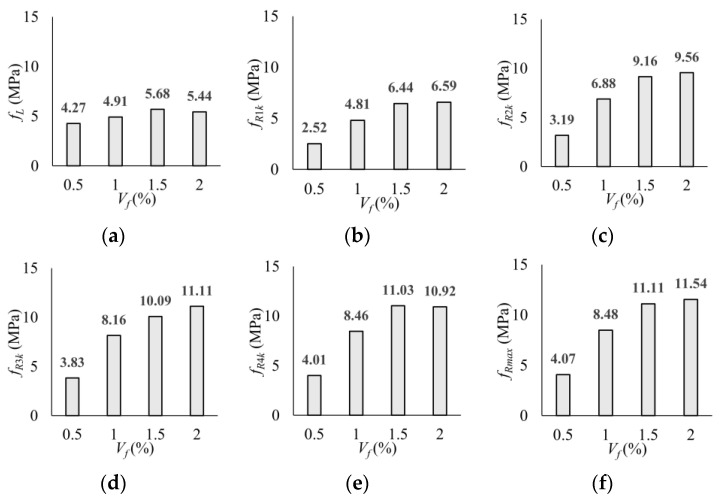
Effect of volumetric fiber ratio on flexural tensile strength: (**a**) *f_L_*; (**b**) *f_R_*_1*k*_; (**c**) *f_R_*_2*k*_; (**d**) *f_R_*_3*k*_; (**e**) *f_R_*_4*k*_; (**f**) *f_R_*_max_.

The variation in the strengths (*f_R_*) at all characteristic points according to the volumetric fiber ratios is graphically presented in Figure 16. A comparison of the strengths corresponding to cracking (*f_L_*) shows that the fiber ratio has no significant effect. The main advantages of employing PP fibers were observed in the post-cracking region through the crack bridging ability of the fibers. It was determined that the strengths are in an increasing trend with the increase in the macro-PP fiber volume ratio (Figure 16). However, there was no significant difference between 1.5% and 2.0% fiber ratios. When comparing the *f_R_*_1*k*_ values, which represent the SLS, the strength increased with increasing fiber content. The increase in strength was quite limited to between 1.5% and 2.0% fiber by volume. Similarly, the strength values for ULS (*f_R_*_3*k*_) increased with fiber content.

#### 3.4.3. Cracking Behavior

**Figure 17:**
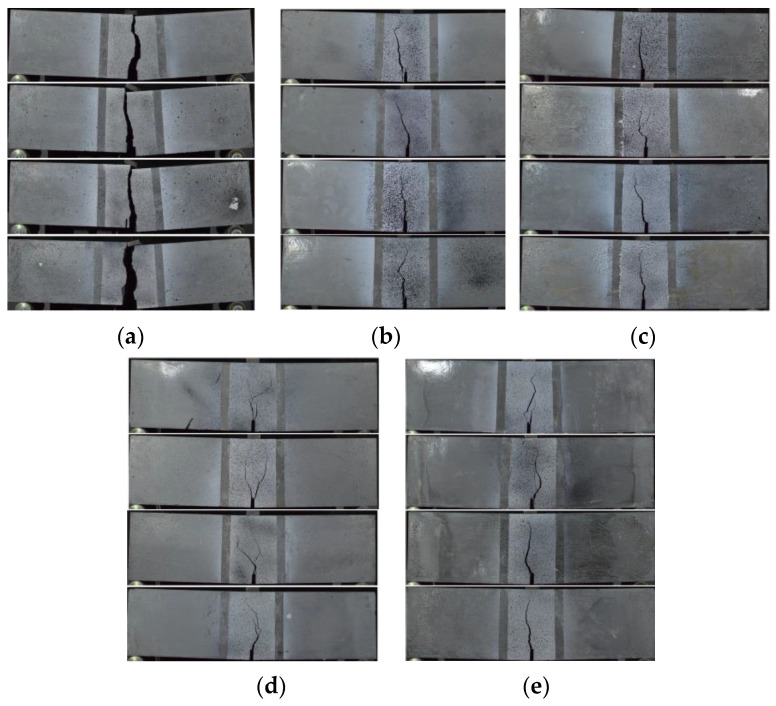
Cracking patterns of the beam specimens: (**a**) *V_f_* = 0%; (**b**) *V_f_* = 0.5%; (**c**) *V_f_* = 1.0%; (**d**) *V_f_* = 1.5%; (**e**) *V_f_* = 2.0%.

**Figure 18:**
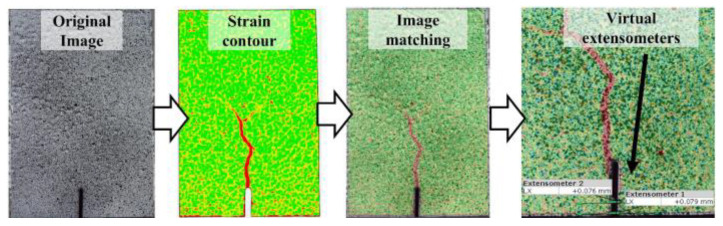
Measurement of the CMOD with GOM correlate [60].

**Figure 19:**
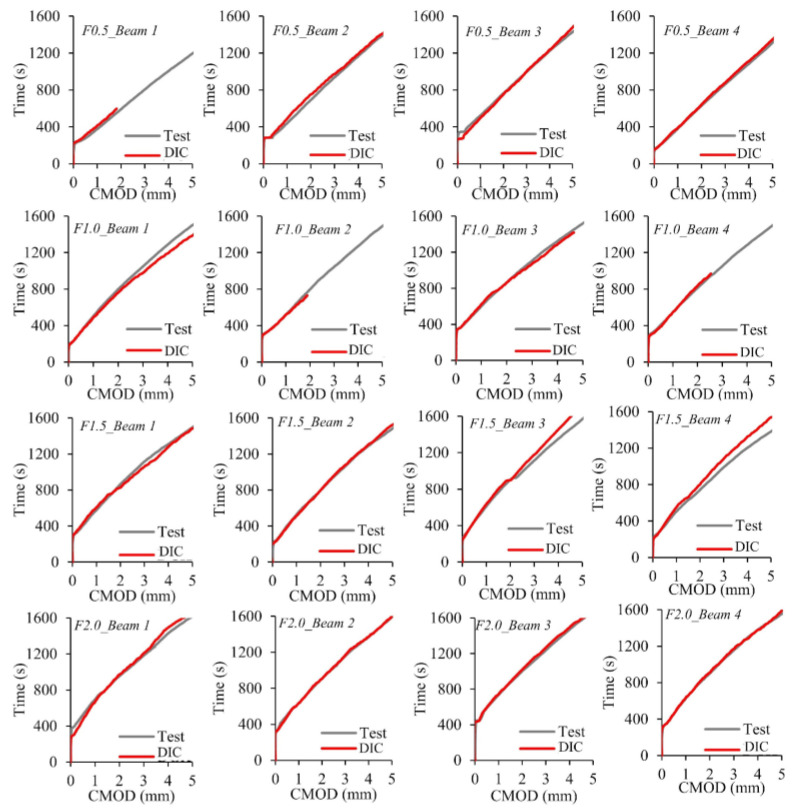
Time–CMOD curves of the test beams.

The cracking patterns observed at the end of the bending tests for each fiber volume ratio are illustrated in Figure 17. All beams exhibited identical cracking behavior, starting at the notch tip and progressing toward the loading point. For the non-fiber beams, failure occurred immediately after cracking in the notch area, causing the specimens to split into two pieces (Figure 17a). Unlike the beams without fibers, the opening and propagation of the cracks were limited due to the crack bridging ability of the macro-PP fibers and sudden failure could be prevented (Figure 17b–e). In this study, GOM Correlate [60] software was utilized to perform two-dimensional digital image correlation (2D-DIC) analysis. With the help of the software, virtual extensometers were placed under the notch, and CMOD values were determined (Figure 18). The measurement accuracy of the 2D-DIC was evaluated using time–CMOD curves consisting of experimental and analysis results (Figure 19). Figure 19 shows that the measurements obtained with 2D-DIC are quite compatible with the experimental results, which confirmed the measurement accuracy of 2D-DIC. The compatibility in the graphics shows that the 2D-DIC can successfully determine the deformations on the specimen surface.

**Figure 20:**
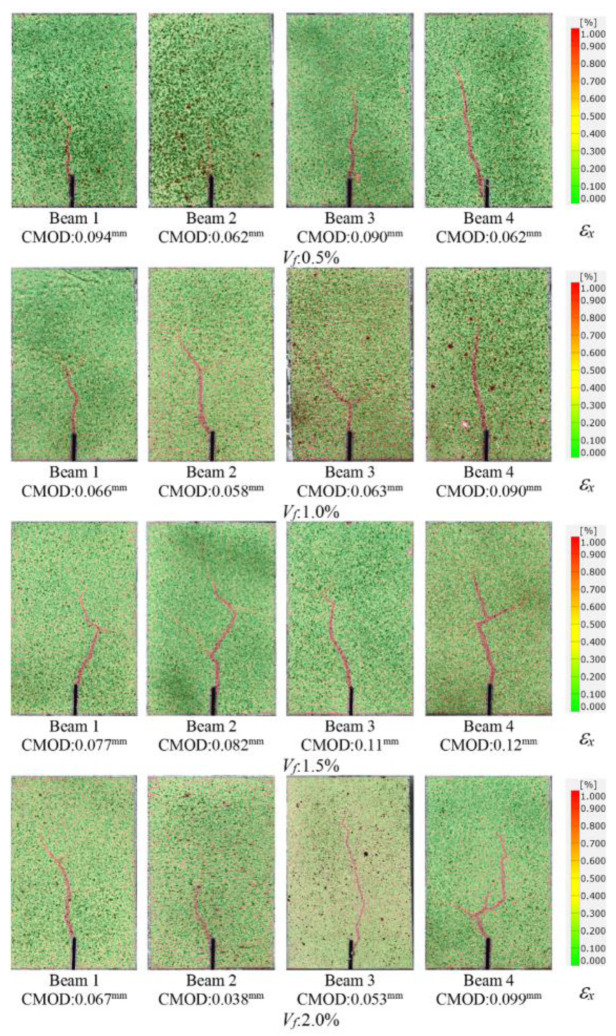
Strain (*ε_x_*) contours of the beams at the cracking stage.

In order to visualize the crack formations, the strains in lateral direction (*ε_x_*) were determined by using GOM Correlate software [60]. The lateral strain (*ε_x_*) contour of the beam specimens with different fiber volume ratios at the cracking stage is presented in Figure 20. As can be seen from Figure 20, the onset of cracking was monitored accurately by 2D-DIC analysis. It was noted that, due to the abrupt nature of cracking, the closest image to the crack initiation can be presented in Figure 20. Although no visible cracks were observed by the naked eye at this stage, the early stages of cracking could be visualized using 2D-DIC.

**Figure 21:**
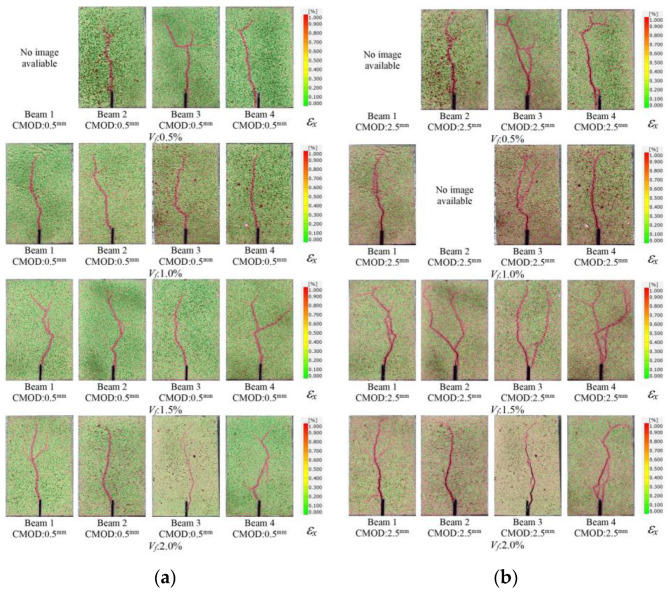
Strain (*ε_x_*) contours of the beams at different limit stages: (**a**) SLS; (**b**) ULS.

To examine the crack propagation for SLS and ULS which correspond to the CMOD values of 0.5 mm and 1.5 mm, respectively, the *ε_x_* contour of the test beams is presented in Figure 21. The propagation of the cracks was found to be similar in all beams for both limit states. There was no clear difference between the specimens with varying amounts of macro-PP fibers.

**Figure 22:**
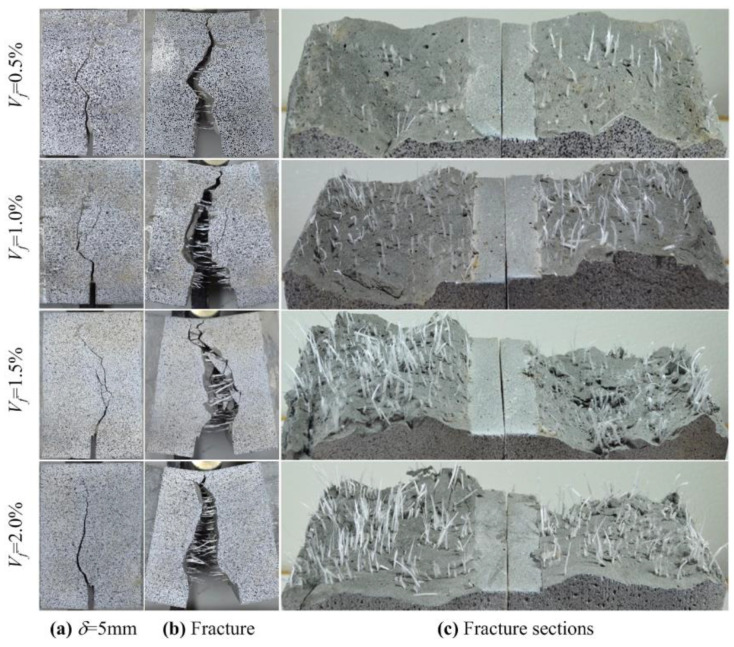
Distribution and orientation of macro-PP fibers in the fracture sections.

To investigate the role of macro-PP fibers on the fracture behavior of UHPC beams, representative specimens for each fiber ratio were selected and loaded to failure beyond the 5 mm deflection value at which the standard tests were terminated. The fracture sections of the specimens are presented in Figure 22. Figure 22b clearly shows the crack bridging ability of the macro-PP fibers in the fracture section. Despite the presence of excessive crack openings, the fibers prevented brittle failure through crack bridging followed by pull-out behavior. Significant deformation was observed in the fibers with some instances leading to rupture. The examination of the fracture sections in Figure 22c reveals a generally good fiber distribution. The deformations that occur in the fibers during debonding were also visible.

## 4. Tensile Constitutive Law Based on Model Code 2020

Post-cracking tensile behavior is considered to be the most important feature of fibrous concrete in structural design. Post-cracking tensile behavior can be directly determined by uniaxial tensile tests. The stress–strain (*σ*-*ε*) and stress–crack opening (*σ*-*w*) relations of fibrous concrete are obtained by using specimens in the shape of dog-bone, prism or notched prism. However, uniaxial tensile tests are difficult to perform, error-prone, and time-consuming [71,72]. Due to their ease of application, indirect tensile tests (splitting tensile, flexural, etc.) are widely used for fibrous concrete. Flexural tests on notched prismatic beams, where the load–crack opening relation is obtained, are one of the most commonly used test methods for obtaining post-cracking behavior. However, the data collected from flexural tests do not represent the actual stress–strain relation of the material and simplified transformations or inverse analysis methods should be applied [72,73]. According to MC2020 [60], the flexural tensile strengths (*f_R_*) corresponding to specific crack mouth opening displacement (CMOD) values are used to derive the simplified post-cracking stress-strain law.

**Figure 23:**
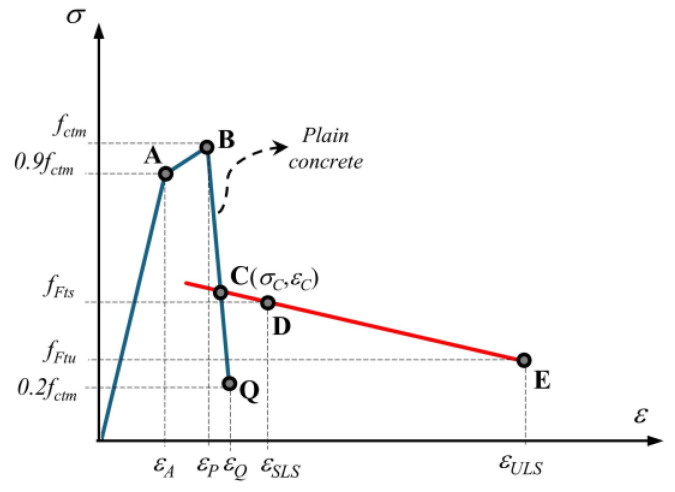
Stress–strain law for the softening behavior of FRC (Case I) in MC2020 [52].

In MC2020 [60], different levels of approximation (LoA) are considered to stress–crack opening and stress–strain laws in uniaxial tension for the post-cracking behavior of fiber reinforced concrete (FRC). In the third level of approximation (LoA III), four cases consist of two softening and two hardening behaviors considered for stress-strain law. FRC is classified as softening material (Case I) if the serviceability residual strength (*f_Fts_*) is equal to or less than 0.8*f_ctm_*. The *f_Fts_* value is determined by Equation (4). The same constitutive relationship as that of plain concrete is used in uniaxial tension up to the peak strength *f_ctm_* (Figure 23). The *f_ctm_* is determined by Equation (5) using the characteristic compressive strength (*f_ck_*), which is obtained by the relationship in Equation (6). For the mean compressive strength (*f_cm_*), the strengths obtained from 100 mm cubic specimens were converted to 150 × 300 mm cylinder strength by applying the conversion proposed in Riedel et al. [74] (Table 3). It is noted that the stress–strain law for all mixtures was in accordance with Case I as specified in MC2020 [60], as shown in Table 3.

A bilinear relationship was applied to the post-cracking stage for Case I (Figure 23). The post-peak propagation branch BC is analytically described with Equations (7) and (8), where *G_f_* is the fracture energy of the plain concrete is determined by Equation (9). The modulus of elasticity *E_c_* is calculated by Equation (10) when the experimental compressive strength *f_cm_* is known. The *α_E_* is 1.0 for quartzite aggregate and *E_c0_* is 21.5 × 10^3^ MPa in Equation (10). The strain values corresponding to SLS (*ε_SLS_*) and ULS (*ε_ULS_*) are determined by Equations (11) and (12), respectively. In these equations, ultimate tensile strain *ε_Fu_* is equal to 0.02 for variable strain distribution along the cross-section, and the characteristic structural length *l_cs_* is taken equal to the beam cross-sectional height in accordance with MC2020 [60]. The branch DE is defined by two points corresponding to (*ε_SLS_*, *f_Fts_*) and (*ε_ULS_*, *f_Ftu_*). *f_Ftu_* represents the ultimate residual strength and is determined by Equation (13) where *w_u_* is the maximum crack opening taken as 2.5 mm. Point C is obtained geometrically by the intersection of branch BQ and DE (Figure 23). Table 4 shows the coordinates of the characteristic points on the stress–strain diagrams.

(4) $$f_{Fts}=0.37f_{R1k}\;\;(MPa)$$

(5) $$f_{ctm}=1.8\ln\left(f_{ck}\right)-3.1\;\;(MPa)$$

(6) $$f_{cm}=f_{ck}+8\;(MPa)$$

(7) $$\frac{\sigma -f_{ctm}}{{0.2f}_{ctm}-f_{ctm}}=\frac{\varepsilon -\varepsilon_{P}}{\varepsilon_{Q}-\varepsilon_{P}}\;for\;\varepsilon_{p}\leq \varepsilon \leq \varepsilon_{c}$$

(8) $$\varepsilon_{Q}=\frac{G_{F}}{f_{ctm}\cdot l_{cs}}+\left(\varepsilon_{P}-\frac{0.8\cdot f_{ctm}}{E_{c}}\right)$$

(9) $$G_{f}=85f_{ck}^{0.15}\;\;(N/m)$$

(10) $$E_{c}=E_{c0}\cdot \alpha_{E}{\left(\frac{f_{cm}}{10}\right)}^{1/3}\;\;(MPa)$$

(11) $$\varepsilon_{SLS}={CMOD}_{1}/l_{cs}$$

(12) $$\varepsilon_{ULS}=w_{u}/l_{cs}=min\left(\varepsilon_{Fu};2.5/l_{cs}\right)$$

(13) $$f_{Ftu}=f_{Fts}-\frac{w_{u}}{{CMOD}_{3}}\left(f_{Fts}-0.57f_{R3k}+0.26f_{R1k}\right)\geq 0\;\;(MPa)$$

**Figure 24:**
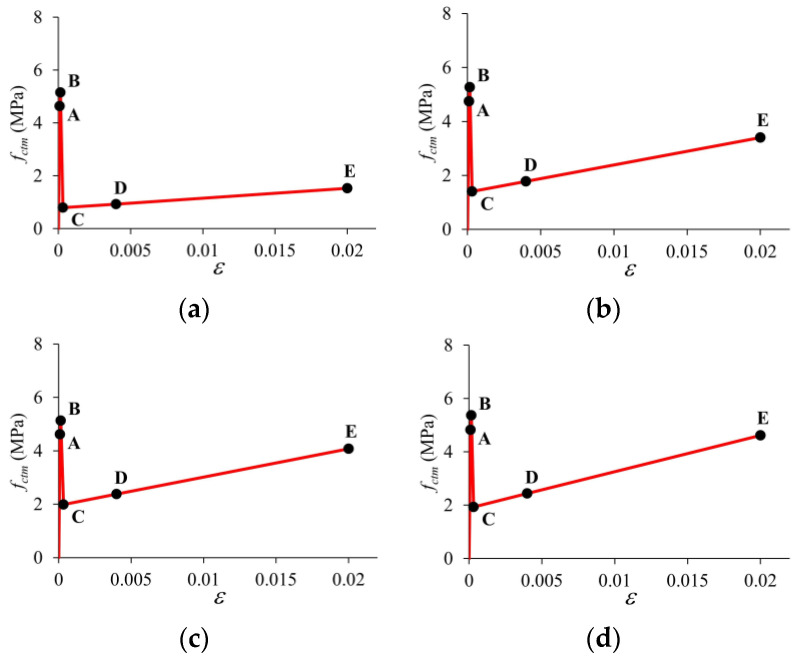
Tensile stress–strain laws obtained by inverse analysis for different volumetric fiber ratios: (**a**) *V_f_* = 0.5%; (**b**) *V_f_* = 1.0%; (**c**) *V_f_* = 1.5%; (**d**) *V_f_* = 2.0%.

The predicted tensile stress–strain laws for each macro-PP fiber dosage are presented in Figure 24. The analysis revealed stress–strain behavior characterized by strain softening for all fiber ratios (Figure 24). Although a limited increase in cracking strength was obtained with increasing fiber content, fiber volume ratios of 1.5% and 2.0% proved to be very effective in the post-cracking stage (Figure 24). Compared to the F0.5, the F1.5 and F2.0 mixtures 2.0% exhibited strength increases of 2.7 and 3.0 times, respectively. Similar behavior has been observed in recent experimental studies on UHPC with PP fibers. Uniaxial tensile tests conducted by Lin et al. [45] revealed that UHPC specimens containing 3% by volume of PP fibers exhibited strain softening behavior, with a corresponding cracking strength of 6.10 MPa. Chen et al. [75] obtained softening behavior in uniaxial tensile tests of dog bone-shaped specimens containing 3 vol% PP fiber with initial crack and post-cracking strengths of 6.8 MPa and 3.57 MPa, respectively. However macro-PP fibers had a significant impact on the post-cracking stage.

## 5. Discussion

Macro-PP fibers significantly affected the workability of UHPC. For fiber contents above a 0.5% volume, a fluidity reduction of up to 50% was observed. Similar findings related to the increase in PP fiber content in UHPC have been reported by other researchers. According to Li et al. [39], the flowability of UHPC increased with an increasing PP fiber diameter, and decreased with increasing fiber content and length. Shen et al. [42] found that fluidity of UHPC decreased by 8.0%, 15.3%, and 24.7% for PP fiber content of 1.0%, 2.0% and 3.0% compared to non-fiber mixture, respectively. The same trend was also reported by Yan et al. [37] for increasing micro-PP fiber ratios.

The compressive strength of the mixtures with macro-PP fiber increased by up to 13% compared to the non-fiber mixture, while no significant effect was observed in tensile strength. Similar results were obtained regarding compressive strength in previous studies on the use of PP fiber in UHPC. In Lin et al. [45], the use of macro-PP fiber at 3.0% by volume did not change the compressive strength compared to UHPC without fiber, while He et al. [41] (2021) found increases of 6.9% and 13.8% with 2.0% high-performance macro-PP fiber compared to 1.0% and 1.5%, respectively. In the case of UHPC with micro-PP fibers, the compressive strength decreases with an increasing fiber content (Shen et al., 2023; Yan et al., 2021) [37,42]. In the study by Yan et al. [37], micro-PP fibers showed only a slight increase in compressive strength at a 0.5% fiber ratio by volume compared to the non-fiber condition. In comparison, no significant effect was observed for 1.0%, 1.5%, and 2.0% fiber ratios. A notable decline in strength was observed when the fiber content was increased to 2.5%. Here, the inefficiency of PP fibers in compressive strength can be attributed to an uneven fiber distribution and agglomeration due to reduced fluidity which leads to the formation of weak zones in the concrete matrix. In addition, the low modulus of elasticity of PP fibers is another reason. However, despite the negative effects of reduced workability, increasing fiber content offset these adverse effects and even provided a limited increase in strength.

No significant effect on the splitting tensile strength was found for the macro-PP fiber ratios examined in the study. On the other hand, the effect of the use of micro-PP fibers on the increase of the splitting tensile strength of the UHPC has been reported in previous studies [42,58]. Shen et al. [42] demonstrated that using micro-PP fiber led to an 11.2% increase in splitting tensile strength for the mixture with 2% fiber compared to that without fiber. This increase was reduced to 9.2% when the fiber content was increased to 3%. Al-Mawnes et al. [58] reported an up to 30% increase in splitting tensile strength with 1.5% micro-PP fiber by volume. However, based on a limited number of studies, micro-PP fibers appear to be more effective in controlling micro cracks than long fibers. Nevertheless, it is evident that macro-PP fiber can be employed without compromising strength. In their study, Al-Mawnes et al. [58] obtained significant improvement in both fresh concrete and mechanical properties by increasing the amount of water-reducing admixture. The workability of the mixtures considered in this study can be enhanced by increasing the dosage of admixture, which was originally optimized for non-fiber UHPC mixture, to accommodate higher fiber ratios. This adjustment is expected to positively affect both the compressive and tensile properties.

In this study, macro-PP fiber was found to be highly effective in enhancing both strength and ductility of post-cracking flexural behavior. Regarding the fiber content, volumetric fiber ratios of 1.5% and 2.0% exhibited similar performance. Neira Medina et al. [44] found no significant difference in strength corresponding to the limit of proportionality for macro-PP fiber ratios of 1.0% and 2.0% by volume compared to the UHPC mixture without fiber. On the other hand, using 2% fiber increased the maximum flexural strength by 13%, and deflection hardening behavior was obtained. In studies using micro-PP fibers, the flexural strength of UHPC increased significantly with an increasing volumetric fiber ratio [37,58]. In contrast, Shen et al. [42] reported a decrease in flexural strength with increasing micro-PP fiber content. However, as presented in this study as well as in other studies, the use of PP fiber greatly improved the post-crack behavior and consequently the ductility of UHPC. Examinations of the fracture sections revealed that the macro-PP fibers exhibited strong performance until failure due to their long length. The deformations observed in the fibers indicate that the fibers provide effective pull-out behavior. Consequently, significant increases in strength and energy absorption capacity were achieved.

## 6. Conclusions

This study investigates the effects of macro-polypropylene (PP) fiber on the mechanical properties of ultra-high-performance concrete (UHPC) in terms of compressive strength, splitting tensile strength, flexural tensile strength, and cracking behavior. The tensile stress–strain law was also determined by inverse analysis proposed in MC2020 using bending test results. The main conclusions are as follows:

1. It is seen that the fluidity of UHPC decreased with the increase in macro-PP fiber content, as expected. Using 2% fiber by volume is significantly reduced by nearly half compared to the case without fiber.
2. No significant difference in compressive strength for mixtures except for the mixture containing macro-PP fiber 2.0% by volume. With a fiber content of 2%, an increase of about 13% was achieved compared to the non-fiber mixture. It was also seen that the use of macro-PP fiber had no significant contribution in splitting tensile strength.
3. The increasing dosage of macro-PP fibers has a limited effect on the cracking strength. However, the incorporation of macro-PP fibers into UHPC led to a notable improvement in post-cracking behavior and, consequently, an enhancement of the overall energy absorption capacity. While deflection hardening behavior was obtained for all ratios except 0.5, a 25% increase in post-cracking strength was achieved in the mixture containing 2% fiber by volume.
4. The non-fiber beams fractured immediately after cracking in the notch area, causing the specimens to split into two pieces. Unlike beams without fibers, the opening and propagation of cracks were limited by the crack bridging ability of the macro-PP fibers, and sudden failure could be avoided. There was no clear difference between the specimens with varying amounts of macro-PP fibers. In addition, the initial cracking stage was monitored accurately by 2D-DIC analysis. Examination of the fracture sections revealed a generally uniform fiber distribution.
5. According to the tensile stress–strain law obtained by the inverse analysis proposed in MC2020, it was determined that the contribution of macro-PP fiber use to tensile strength was quite limited. However, it was observed that the increasing amount of the macro-PP fibers significantly affected the post-crack ductility.

According to our overall conclusions, the use of macro-PP fiber has the potential to provide significant benefits in the residual flexural strength and fracture energy of UHPC. It was determined that the use of macro-PP fiber can provide effective crack control. However, macro-PP fiber showed a limited effect on compressive and tensile strength. Future studies can be conducted to investigate the effects of PP fibers with different geometric properties on UHPC. In this context, future studies are needed to explore the influence of polypropylene fibers with varying geometric properties such as length, diameter, and surface texture for enhancing the mechanical properties of UHPC. Detailed investigations including stress–strain relationships of compressive and tensile behaviors will provide deeper insights into the structural behavior. In addition, SEM analyses should be performed for a detailed understanding of the fracture behavior of UHPC with macro-PP fibers.

## Figures and Tables

**Table 1:** Materials and proportions of the UHPC mixtures (kg/m^3^).

Material	NF	F0.5	F1.0	F1.5	F2.0
Cement	700	700	700	700	700
Silica fume	170	170	170	170	170
Blast furnace slag	300	300	300	300	300
Aggregate	1032	1015	1005	992	978
Water	200	200	200	200	200
Admixture	17	17	17	17.4	17.8
Fiber	0	4.5	9	13.5	18
Fiber (by volume)	0%	0.5%	1.0%	1.5%	2.0%

**Table 2:** Average flexural tensile strengths of the UHPC mixtures.

Mixture	*f_L_* *	*f_R_*_1*k*_ *	*f_R_*_2*k*_ *	*f_R_*_3*k*_ *	*f_R_*_4*k*_ *	*f_R,min_* *	*f_R,max_* *
NF	4.89	-	-	-	-	-	-
F0.5	4.27	2.52	3.19	3.83	4.01	2.39	4.07
F1.0	4.91	4.81	6.88	8.16	8.46	4.33	8.48
F1.5	5.68	6.44	9.16	10.09	11.03	5.52	11.11
F2.0	5.44	6.59	9.56	11.11	10.92	5.30	11.54

* values in MPa.

**Table 3:** Mechanical properties of mixtures determined by MC2020 [52].

Mixture	*f_cm_* (MPa)	*E_c_* (MPa)	*G_f_* (N/mm)	*f_ctm_* (MPa)	*f_Fts_* (MPa)	*f_Ftu_* (MPa)	Material Class
F0.5	106	47214	0.17	5.15	0.93	1.53	Softening (Case I)
F1.0	113	48182	0.17	5.27	1.78	3.40	Softening (Case I)
F1.5	105	47072	0.17	5.13	2.38	4.08	Softening (Case I)
F2.0	118	48982	1.72	5.36	2.44	4.62	Softening (Case I)

**Table 4:** Characteristic points expressing stress–strain laws.

Point	F0.5		F1.0		F1.5		F2.0	
	*ε*	*σ* (MPa)	*ε*	*σ* (MPa)	*ε*	*σ* (MPa)	*ε*	*σ* (MPa)
A	0.00010	4.64	0.00010	4.74	0.00010	4.62	0.00010	4.83
B	0.00015	5.15	0.00015	5.27	0.00015	5.13	0.00015	5.36
C	0.00034	0.80	0.00033	1.41	0.00028	1.99	0.00029	1.93
D	0.004	0.93	0.004	1.78	0.004	2.38	0.004	2.44
E	0.02	1.53	0.02	3.40	0.02	4.08	0.02	4.62

## Data Availability

The original contributions presented in this study are included in the article. Further inquiries can be directed to the corresponding author.
